# Modulating the growth of chemically deposited ZnO nanowires and the formation of nitrogen- and hydrogen-related defects using pH adjustment†

**DOI:** 10.1039/d1na00785h

**Published:** 2022-02-23

**Authors:** José Villafuerte, Eirini Sarigiannidou, Fabrice Donatini, Joseph Kioseoglou, Odette Chaix-Pluchery, Julien Pernot, Vincent Consonni

**Affiliations:** Université Grenoble Alpes, CNRS, Grenoble INP, LMGP F-38000 Grenoble France vincent.consonni@grenoble-inp.fr; Université Grenoble Alpes, CNRS, Grenoble INP, Institut NEEL F-38000 Grenoble France julien.pernot@neel.cnrs.fr; Physics Department, Aristotle University of Thessaloniki 54124 Thessaloniki Greece

## Abstract

ZnO nanowires (NWs) grown by chemical bath deposition (CBD) have received great interest for nanoscale engineering devices, but their formation in aqueous solution containing many impurities needs to be carefully addressed. In particular, the pH of the CBD solution and its effect on the formation mechanisms of ZnO NWs and of nitrogen- and hydrogen-related defects in their center are still unexplored. By adjusting its value in a low- and high-pH region, we show the latent evolution of the morphological and optical properties of ZnO NWs, as well as the modulated incorporation of nitrogen- and hydrogen-related defects in their center using Raman and cathodoluminescence spectroscopy. The increase in pH is related to the increase in the oxygen chemical potential (*μ*_O_), for which the formation energy of hydrogen in bond-centered sites (H_BC_) and V_Zn_-N_O_-H defect complexes is found to be unchanged, whereas the formation energy of zinc vacancy (V_Zn_) and zinc vacancy-hydrogen (V_Zn_-*n*H) complexes steadily decreases as shown from density-functional theory calculations. Revealing that these V_Zn_-related defects are energetically favorable to form as *μ*_O_ is increased, ZnO NWs grown in the high-pH region are found to exhibit a higher density of V_Zn_-*n*H defect complexes than ZnO NWs grown in the low-pH region. Annealing at 450 °C under an oxygen atmosphere helps tuning the optical properties of ZnO NWs by reducing the density of H_BC_ and V_Zn_-related defects, while activating the formation of V_Zn_-N_O_-H defect complexes. These findings show the influence of pH on the nature of Zn(ii) species, the electrostatic interactions between these species and ZnO NW surfaces, and the formation energy of the involved defects. They emphasize the crucial role of the pH of the CBD solution and open new possibilities for simultaneously engineering the morphology of ZnO NWs and the formation of nitrogen- and hydrogen-related defects.

## Abstract 2

The effects of pH on the chemical bath deposition of ZnO nanowires are investigated in detail with a special emphasis on the physicochemical processes in solution and the incorporation of nitrogen- and hydrogen-related defects.

## Graphical abstract

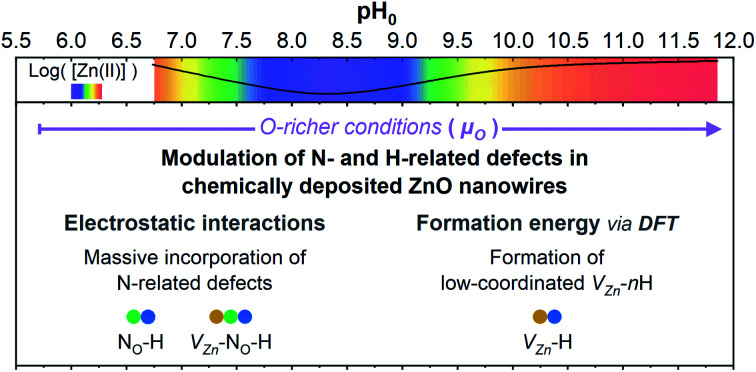

## 1. Introduction

The study of ZnO nanowires (NWs) in the last two decades has shown high interest towards gaining a better understanding on their potential use as efficient nanoscale devices in the fields of biological and/or chemical sensing,^1^ and optoelectronic,^2^ photovoltaic,^3^ piezotronic,^4^ and piezoelectric^5^ applications. ZnO NWs also possess the advantage to be grown by the low-temperature and low-cost solution route of chemical bath deposition (CBD). Their crystallization follows the thermally-activated dehydration of [Zn(H_2_O)_6_]^2+^ ions coming from Zn(ii) ions representing the limiting reactant at low pH, and their growth is kinetically controlled by the thermally-activated decomposition of hexamethylenetetramine (HMTA) that slowly releases hydroxide ions in an aqueous solution.^6–10^ Hence, the different experimental parameters such as the chemical precursor concentration,^8,9,11,12^ nature of the species present in the solution,^11,13–17^ pH of the solution,^11,18,19^ temperature and growth time,^10,20^ have shown to play a key role in the morphological, structural, chemical, optical, and electrical properties of ZnO NWs. Universally, hydronium (H_3_O^+^) and hydroxide (OH^−^) ions are referred to as the water ions, and their study is crucial in aqueous solution. In the case of the present study, special attention is paid to the physicochemical parameter of pH. The pH scale measures how acidic or basic a solution is, and as stated in simple terms, the pH operational concept is defined as:

1 pH = −log_10_[H_3_O^+^]

where [H_3_O^+^] is the hydronium ion molar concentration of a solution with respect to a reference pH value.^21–23^ Conversely, the hydroxide ion molar concentration [OH^−^] is also derived from pH measurements and immediately highlights the importance of pH in the CBD solution as a potential source of oxygen for the crystallization of ZnO NWs. Additionally, it is well-known that pH also impacts the growth mechanisms of ZnO NWs,^10,11,19^ and the concentration and nature of the species present in the CBD solution.^9^ By the addition of ammonium chloride (NH_4_Cl), Joo *et al.* showed that in a zinc-only CBD system, a modest variation (<1.5-fold range) of the aspect ratio of ZnO NWs can be expected by adjusting the pH alone within the range of 10.6 to 11.4.^11^ Dahiya *et al.* showed that the addition of ammonium hydroxide (NH_4_OH) slowly degrades the optical response of ZnO NWs through the increase in the concentration of point defects within the pH range of 6.6 to 7.1.^18^ Additionally, the intentional doping of ZnO NWs with Al,^15^ Ga,^13^ and Cu,^14^ has been developed through the addition of their respective chemical precursors into the CBD solution with different amounts of ammonia (NH_3_) within the pH range of ∼7 to ∼11.

From a theoretical approach, the formation of pure bulk ZnO should satisfy the following relation:

2 *μ*^s^_ZnO_ = *μ*_Zn_ + *μ*_O_

where *μ*^s^_ZnO_, *μ*_Zn_ and *μ*_O_ represent the chemical potentials of bulk ZnO in the wurtzite phase, Zn atoms, and O atoms, respectively.^24,25^ Experimentally, the oxygen chemical potential *μ*_O_ depends on the growth conditions, which can be either Zn-rich, O-rich, or intermediate. *Via* density functional theory (DFT) calculations, it has been shown that the dependence of the formation energy of point defects in ZnO with respect to *μ*_O_, *e.g.* a Zn-rich or O-rich environment, would lead to the formation of oxygen or zinc vacancies, respectively.^25,26^ Simultaneously, regarding the growth of ZnO *via* solution methods, the pH also has a relationship with *μ*_O_,^27,28^ which consequently affects the formation energy of intrinsic and extrinsic point defects.^29^ Through the paramount contribution of Van de Walle,^30^ it was revealed from DFT calculations that interstitial hydrogen (H_i_) systematically acts as a shallow donor over the expected range of Fermi level, which is different from the typical amphoteric behavior that hydrogen impurities exhibit in other semiconductors. Consequently, hydrogen-related defects are defined as H_i_ in the bond-centered site (H_BC_), acting as a shallow donor,^30^ substitutional hydrogen on the oxygen lattice site (H_O_), acting as a shallow donor,^25^ zinc vacancy-hydrogen (V_Zn_-*n*H) complexes, where *n* represents the number of involved H_i_ atoms,^31,32^ and V_Zn_-H, V_Zn_-2H, and V_Zn_-3H act as a deep acceptor, a neutral defect, and a shallow donor, respectively.^33^ It is worth mentioning that it has recently been shown that the V_Zn_-3H defect complex has a lower formation energy than H_BC_, which had previously been presumed to be the most stable configuration.^33^*Via* four-point probe resistivity measurements, the high free electron density in the range of 2.7 × 10^18^ to 3.1 × 10^19^ cm^−3^ for spontaneously grown ZnO NWs and 6.4 × 10^17^ to 1.1 × 10^19^ cm^−3^ for selective-area-grown ZnO nanorods has directly been attributed to the high concentration of H_BC_, V_Zn_-3H, and H_O_ defects, through complementary Raman and 5 K cathodoluminescence spectroscopy measurements.^33,34^ A more recent study also showed by DFT that the V_Zn_-N_O_-H defect complex acts as a deep acceptor with a relatively low formation energy and how the nature and concentration of the nitrogen- and hydrogen-related defects are engineered using thermal annealing under an oxygen atmosphere.^35^ The thermal annealing of ZnO NWs at 300 °C promotes a reduction of the free electron density from 10.2 × 10^17^ to 5.6 × 10^17^ cm^−3^, following the analysis of longitudinal optical phonon-plasmon (LPP) coupling; additionally, the activation mechanism of the V_Zn_-N_O_-H defect complex at 500 °C is favored through the combination of residual N_O_-H defects with V_Zn_. Hence, with advances in the understanding of the nitrogen and hydrogen-related defects in unintentionally doped ZnO NWs grown by CBD,^33,35^ it is valuable to further understand how the pH of the CBD solution influences the formation and incorporation of these defects.

In the present study, ZnO NW arrays are initially grown using a series of CBD solutions within the pH range of 7.00 to 11.07 and subsequently annealed thermally at 450 °C under an oxygen atmosphere, following the methodology used in ref. 35. A deep understanding and fine evolution of nitrogen- and hydrogen-related defects is given from the combination of *in situ* pH measurements, thermodynamic computations, Raman and 5 K cathodoluminescence spectroscopy with DFT calculations. Following this comprehensive analysis, the latent opportunity for engineering the optical and electrical properties of ZnO NWs *via* the modification of pH in the CBD solution is unveiled.

## 2. Experimental and theoretical methods

### 2.1 Synthesis and thermal annealing of ZnO nanowires

Silicon (100) and Corning glass substrates were cleaned with acetone and isopropyl alcohol in an ultrasonic bath to remove residual dust particles and organic contaminants. The ZnO sol–gel solutions containing 375 mM zinc acetate dihydrate (Zn(CH_3_COO)_2_·2H_2_O, Sigma-Aldrich) and 375 mM monoethanolamine (MEA, Sigma-Aldrich) were mixed in pure ethanol and stirred on a hot plate for several hours at 60 °C and then at room temperature to assure the proper dissolution of Zn(CH_3_COO)_2_·2H_2_O. The substrates were mechanically dipped into the sol–gel solution and gently removed under a controlled atmosphere (<15% hygrometry). Subsequently, they were annealed at 300 °C for 10 min on a hot plate to remove residual organic compounds and at 500 °C for 1 h in an oven under air for the crystallization of the ZnO seed layer. ZnO NWs were grown by CBD in a sealed reactor at 85 °C containing deionized water-based solutions of zinc nitrate hexahydrate (Zn(NO_3_)_2_·6H_2_O, Sigma-Aldrich) and HMTA (C_6_H_12_N_4_, Sigma-Aldrich) mixed in an equimolar ratio of 30 mM. Additionally, the initial pH of the solution before heating, denoted as pH_0_, was changed from 7.00 to 11.07 by the further addition of different concentrations of ammonia (NH_3_, Sigma-Aldrich) ranging from 0 to 1505 mM. Table 1 summarizes the concentration of added ammonia and the growth time for each pH_0_ condition. The growth time was varied in the range of 60–360 min to form ZnO NWs with a length higher than 1 μm regardless of the pH_0_ value, which is favorable to investigate the pH effects mainly during the elongation process. A thermal annealing at 450 °C for 1 h was eventually performed in a tubular furnace under an oxygen atmosphere.

**Table 1:** CBD conditions for the series of ZnO NWs grown with different pH_0_ values

Sample	pH_0_	[NH_3_] added (mM)	Growth time (min)
1	7.00	0	360
2	7.20	10	360
3	7.25	20	360
4	7.44	58	360
5	10.55	510	180
6	10.62	580	180
7	10.70	660	90
8	10.76	740	60
9	10.80	840	60
10	10.94	950	90
11	11.07	1505	240

### 2.2 Characterization techniques

During the ZnO NW growth, the *in situ* measurement of pH and temperature was performed with an InLab Versatile Pro pH electrode from Mettler Toledo. The morphology of ZnO NWs was evaluated with a FEI Quanta 250 FESEM instrument. The nature of nitrogen- and hydrogen-related defects was investigated by Raman and 5 K cathodoluminescence spectroscopy. The Raman spectroscopy of ZnO NWs was performed with a Horiba/Jobin Yvon Labram spectrometer equipped with a liquid nitrogen-cooled CCD detector. A 514.5 nm Ar^+^ laser with a power on the sample surface of ∼0.51 mW was focused to a spot size of ∼1 μm^2^ using a 100× objective. The integration time was 200 s per spectral window from 50 cm^−1^ to 3750 cm^−1^. A silicon reference sample was used for spectral calibration at room temperature, with the theoretical Raman line set to 520.7 cm^−1^. The Raman spectral acquisition was achieved in a *z*(−,−)*z̄* geometry, where the laser probes an array of ZnO NWs from the top.

The 5 K cathodoluminescence spectroscopy was performed with a FEI Inspect F50 FESEM instrument equipped with a liquid helium-cooled stage. A low acceleration voltage of 5 kV and a small spot size (*i.e.*, less than 10 nm) were used to probe an array of ZnO NWs from the top. A parabolic mirror controlled with nanomanipulators helped to collect the cathodoluminescence signal on a 550 mm focal length monochromator equipped with a 600 grooves per mm diffraction grating. Cathodoluminescence spectra were acquired with the electron beam irradiating the array of ZnO NWs in scanning mode with an area size of ∼0.50 μm^2^ and recorded with a thermoelectric cooled silicon CCD detector. The 5 K cathodoluminescence acquisition was achieved on several frames of ZnO NWs for each pH_0_ value.

### 2.3 Thermodynamic computations

Thermodynamic simulations were performed with Visual MINTEQ software to determine the speciation diagrams of Zn(ii) species as well as the theoretical solubility plots of ZnO at 85 °C at each NH_3_ concentration. The Zn^2+^ cation in aqueous solution is capable of forming hydroxide and/or amine complexes with two possible ligands (HO^−^ and NH_3_) denoted as L, given the general reactions *n*Zn^2+^ + *i*L ↔ Zn_*n*_L_*i*_^*n*·2+^, where Zn_*n*_L_*i*_^*n*·2+^ is the complex considered with *i* as the coordination number. The related stability constants *β*^L^_*i*_ associated with each reaction are given by 
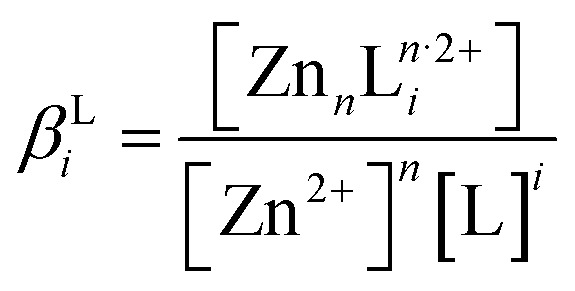
. These constants at room temperature were taken from the National Institute of Standards and Technology (NIST) database and the constants at 85 °C were deduced from the Van't Hoff relation. To calculate the theoretical solubility plots for each condition, Zn-related oxides and hydroxides were considered.

### 2.4 Density-functional theory method

The wurtzite structure of ZnO and the specified point defects from the current study were investigated using the VASP code with projector augmented-wave (PAW) potentials^36,37^ along with the Perdew–Burke–Ernzerhof derivation of the generalized gradient approximation (GGA-PBE)^38,39^ of DFT. The Γ-centered *k*-point mesh defining the reciprocal-space resolution was generated with a Monkhorst-Pack mesh of 8 × 8 × 6 for the 1 × 1 × 1 unit cell and it has a cut-off energy set to 600 eV. The methodology followed in the current investigation was extensively detailed in our previous investigations in ref. 33 and 35. Furthermore, a large size 4 × 4 × 3 supercell of 192 atoms was employed to ensure the structural relaxation and the accurate calculation of these properties of point defects in ZnO. The detailed expression for the formation energy of a defect in a specific charge state (*Q*) is fully developed in ESI† Section 1.

## 3. Results and discussion

### 3.1 Effect of pH of the CBD solution on the growth mechanisms of ZnO NWs

#### 3.1.1 Description of the structural morphology of ZnO NWs

**Fig. 1:**
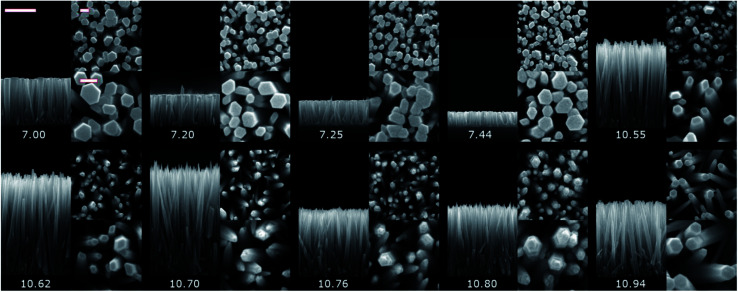
Cross-sectional- and top-view FESEM images of the pristine series of ZnO NWs grown by CBD using pH_0_ values in the range of 7.00–10.94. The scale bars correspond to 1000 nm for the cross-sectional view, and 100 nm for both low magnification and high magnification top-views.

**Fig. 2:**
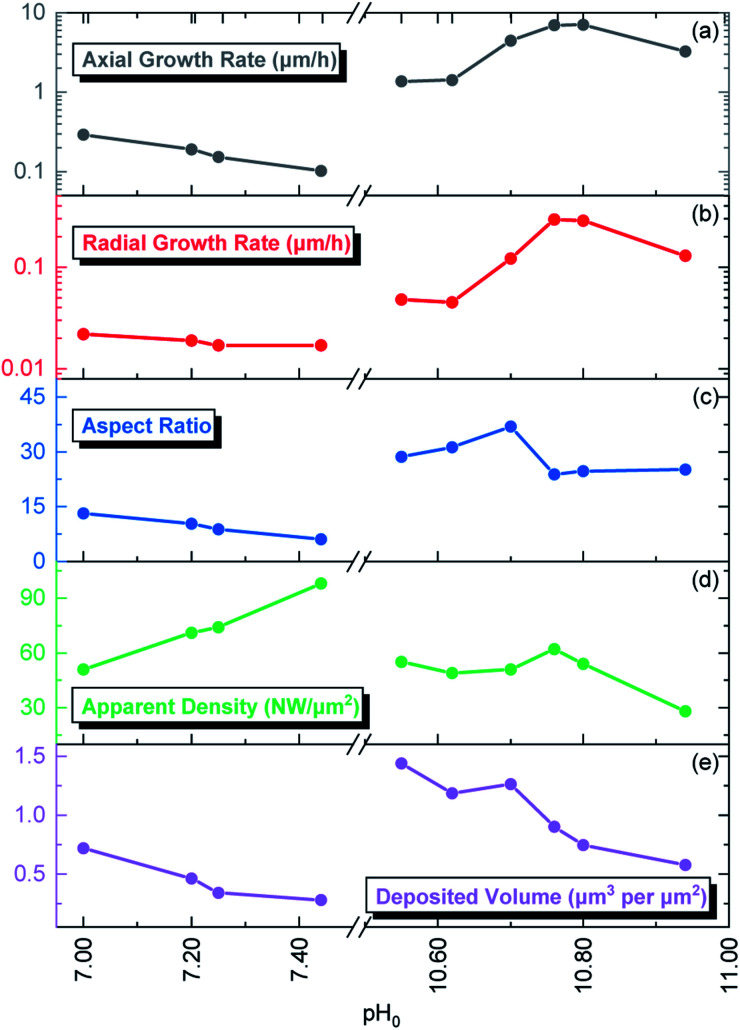
Evolution of the (a) axial growth rate, (b) radial growth rate, (c) aspect ratio, (d) apparent density, and (e) deposited volume of ZnO NWs grown by CBD as a function of pH_0_.

The heterogeneous formation of ZnO NWs was performed on top of the *c*-axis oriented polycrystalline ZnO seed layers deposited *via* the dip-coating technique with the same structural morphology for the entire study.^40,41^ The morphological properties of the as-grown pristine ZnO NWs by CBD with pH_0_ values in the range of 7.00–10.94 are shown in Fig. 1 by FESEM imaging and in Fig. 2 by representing the axial growth rate along the polar *c*-axis, radial growth rate, aspect ratio, apparent density and deposited volume for each condition. The axial and radial growth rates were deduced from the ratio of the length and diameter of ZnO NWs over the effective growth time, respectively. The effective growth time was defined as the total growth time subtracted by the nucleation time of ∼20 min needed to reach the temperature of 65 °C required to start the formation process of ZnO NWs. The deposited volume of ZnO NWs was inferred from their diameter, length, and apparent density. It is worth mentioning here that no growth of ZnO NWs proceeded when using a pH_0_ value of 11.07 as shown in Fig. S2 in the ESI† and the seed layer was even dissolved due to the well-known high solubility of ZnO at high pH.^13^

The pristine ZnO NWs grown in the pH_0_ region of 7.00–10.94 are vertically aligned with the hexagonal shape characteristic of their wurtzite structure oriented along the polar *c*-axis.^42^ The ZnO NWs grown within the increasing pH_0_ region of 7.00–7.44, defined as the low-pH region, exhibit a wide-hexagonal tip despite a drastic decrease in their axial growth rate from 0.29 to 0.10 μm h^−1^, and a correlated significant decrease in their aspect ratio from 13 to 6. Interestingly, the decrease in the axial growth rate is accompanied by an increase in the apparent density of ZnO NWs from 51 to 98 NW per μm^2^. This is attributed to the appearance of O-polar ZnO NWs that have a slower growth rate than their counter Zn-polar ZnO NWs,^40,43^ and hence under standard conditions the Zn-polar NWs grow faster and mask the presence of O-polar NWs. As shown in Table 1, it is evident that no growth was performed along the intermediate pH_0_ region ranging from 7.44 to 10.55 owing to the well-known predominant homogeneous nucleation occurring in the CBD solution.^19^ The ZnO NWs grown within the pH_0_ region of 10.55–10.94, defined as the high-pH region, exhibit higher aspect ratios (>20), which are more suitable for piezoelectric applications.^6,44^ These ZnO NWs have different types of morphologies, where the NWs grown within the pH_0_ range of 10.55–10.62 show a narrow-hexagonal tip while the NWs grown within the pH_0_ range of 10.70–10.80 show a pencil-like tip. These morphologies are correlated with the prominent increase in the axial growth rate of ZnO NWs seen in the high-pH region. ZnO NWs grown with an axial growth rate of ∼1.5 μm h^−1^ have a narrow-hexagonal tip, while ZnO NWs grown with a higher growth rate have a pencil-like tip. These morphologies have previously been shown on ZnO NWs from ref. 10, 13, 14 and 45, using the conditions where no dopant solution was added to the CBD solution with pH_0_ > 10.3. Remarkably, the ZnO NWs grown at a pH_0_ value of 10.94 has a high aspect ratio and they present a narrow-hexagonal tip instead of a pencil-like tip. As shown by Lausecker *et al.*, the drastic increase in the axial growth rate prevents the complete formation of the nonpolar *m*-planes on their sidewalls, hence reducing the size of the NW tip.^14^ Simultaneously, an erosion process of the NW top facets by HO^−^ ions at high pH has also been shown.^46^ However, the extended growths under dynamic conditions cause the depletion of chemical reactants, promoting a decrease in the axial growth rate and giving the opportunity for the nonpolar *m*-planes to form.^10^ Hence, this would explain how our ZnO NWs grown at the pH_0_ value of 10.94 could have passed from a pencil-like tip at the initial growth stage to a narrow-hexagonal tip at the final growth stage.

#### 3.1.2 Effect of pH on CBD solution: thermodynamic simulations *versus* experimental results

Recently, the spontaneous growth of ZnO NWs without the addition of ammonia to the CBD solution has extensively been studied in ref. 10, 33 and 35. Under these standard CBD conditions, an initial equimolar concentration of Zn^2+^ ions, from the solubilization of zinc nitrate, and NH^+^_4_ ions, from the hydrolysis of HMTA, is roughly expected in the bath.^9^ To carefully understand the influence of pH and the behavior of the CBD solution along the growth of ZnO NWs, thermodynamic computations were performed using Visual MINTEQ software. Along these computations, the initial concentration for Zn^2+^ species was set to 30 mM and the concentration of NH^+^_4_ was varied from 30 mM up to 1600 mM, with a 1 mM step. The theoretical pH corresponds to the equilibrium pH in the bath at a given temperature and it was calculated using the software from mass and charge equilibrium balances.

**Fig. 3:**
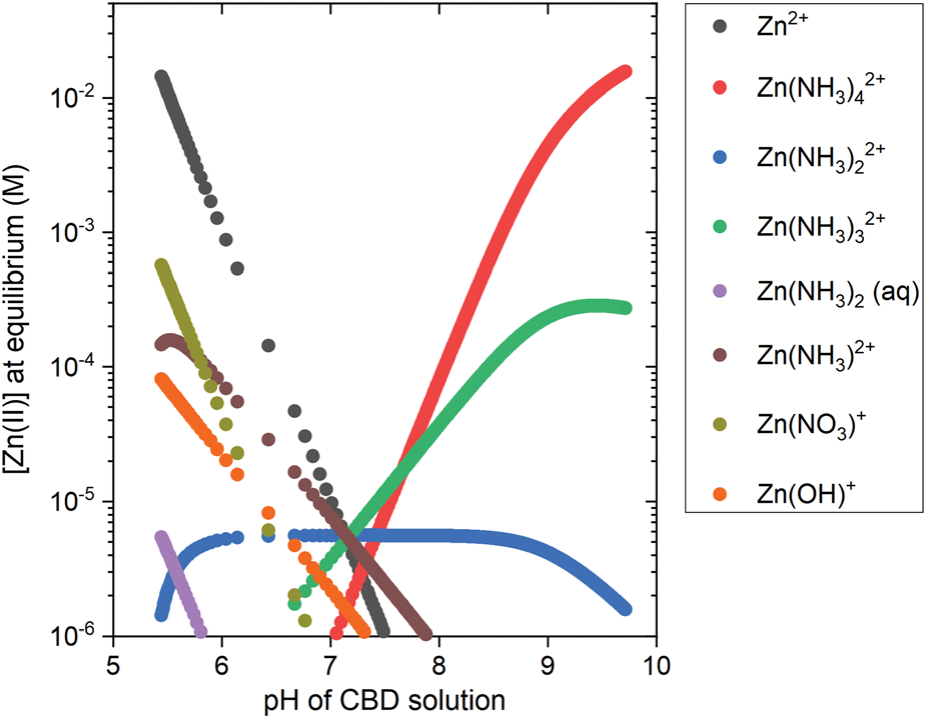
Speciation diagram of Zn(ii) species present in the CBD solution at 85 °C as a function of pH, obtained from the thermodynamic calculations using Visual MINTEQ software. The initial concentration for Zn^2+^ ions was set to 30 mM and the NH_4_^+^ concentration was varied from 30 mM up to 1600 mM, with a 1 mM step.

A speciation diagram of Zn(ii) species at 85 °C is presented in Fig. 3 and shows a crucial difference in the nature of the pH-dependent species: (i) the Zn^2+^ ions are the main species in solution in the low-pH region, whereas (ii) the Zn(ii) amine complexes, including Zn(NH_3_)^2+^_4_ ions as the main species, are predominantly present in solution in the high-pH region.

**Fig. 4:**
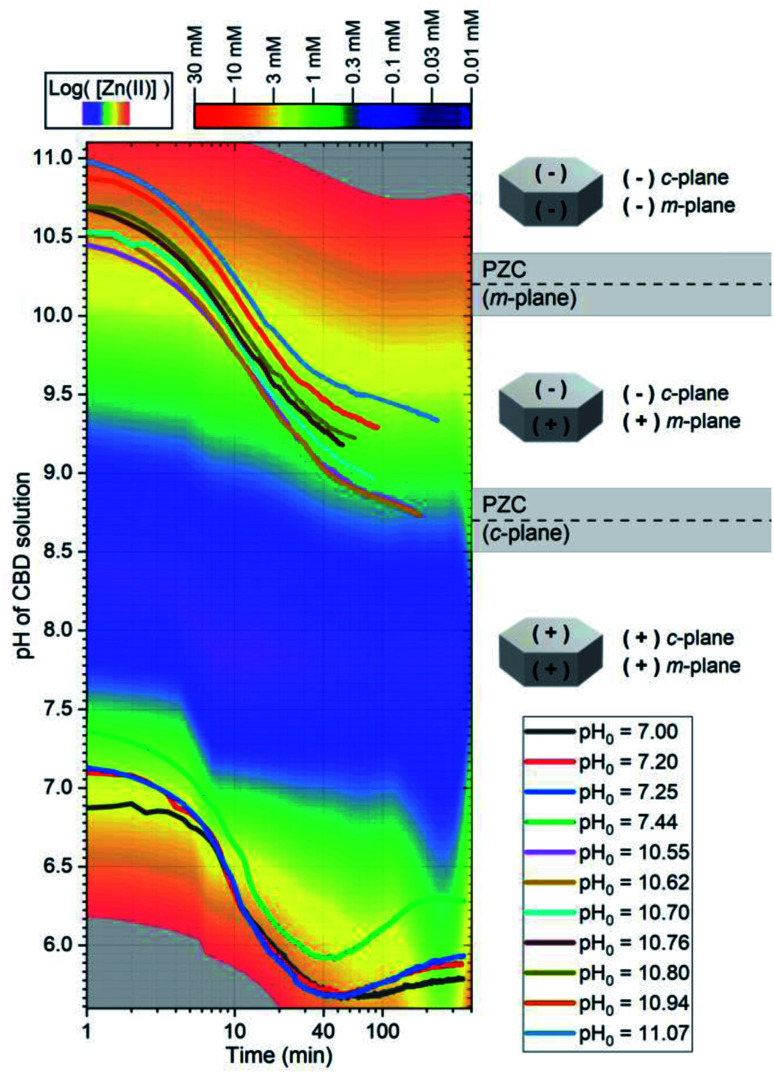
*In situ* measurements of the pH during the CBD of ZnO NWs using pH_0_ values in the range of 7.00–11.07. The solid lines represent the pH of the solution at a given growth time. (i) The color-coded map represents the theoretical equilibrium concentration of soluble Zn(ii) species as calculated using Visual MINTEQ software, and (ii) the schematic diagrams show the expected surface electrical charge of the different crystallographic planes of ZnO NWs according to the PZC values reported in ref. 11, 47, 48 and 49.

To complement the thermodynamic calculations, *in situ* pH and temperature measurements of the CBD solution were performed during the growth of ZnO NWs under different conditions, as detailed in Table 1. Fig. S3(a)† shows that a duration of ∼20 min is required for the growth reactor to reach temperatures >65 °C, which has been shown to be an important starting point for ZnO NW nucleation.^10^ Fig. S3(b)† shows that, as the temperature is increased, a shift to lower values of pH is expected from thermodynamic solubility curves of Zn(ii) species. A whole picture of this growth process is seen in the left section of Fig. 4, where the *in situ* pH measurements are depicted along with the theoretical solubility plots of Zn(ii) species illustrated as a color-coded map.

Specific physicochemical processes and thermodynamic considerations related to surface energy minimization, electrostatic forces, and kinetically-controlled diffusive mechanisms need to be taken into account to understand the growth of ZnO NWs by CBD.^11,50^ In that set of parameters, the supersaturation ratio represents a crucial concept and is defined as the ratio between the initial concentration of Zn^2+^ ions that is equal to 30 mM and the equilibrium concentration of soluble Zn(ii) species.^51^ It should be noted that the growth of ZnO NWs by CBD is typically achieved under intermediate supersaturation conditions, a metastable domain where both heterogeneous and homogeneous growths occur.^6^

The low-pH region shows that increasing the pH_0_ value from 7.00 to 7.44 brings the growth of ZnO NWs to increasing supersaturation levels, which are detrimental for the heterogeneous nucleation; hence, this explains the steady decrease in the axial growth rate and deposited volume of these NWs in Fig. 1 and 2, respectively. The increase in the pH value seen around a growth time of ∼60 min has been attributed to the continuous hydrolysis of HMTA, which releases a large excess of HO^−^ ions in the bath.^9^ Additionally, this supports well the deposition mechanism discussed by D. Lincot, where Case 1 deposition process is associated with a pH shift to higher values.^6^

As shown in the right section of Fig. 4, the surface electrical charge of ZnO NWs is pH dependent and it can show different regions depending on the expected value of the point-of-zero-charge (PZC). The PZC is defined as the point where the surface electrical charge is neutral and does not bear any individual charges. It has been reported to be located at 8.7 ± 0.2 and 10.2 ± 0.2 for the polar *c*-plane and nonpolar *m*-plane of ZnO, respectively.^11,47–49^ All the growths in the low-pH region occur when both the *c*- and *m*-planes are positively charged, and where the dehydration of [Zn(H_2_O)_6_]^2+^ ions and crystallization of ZnO NWs are promoted by the capping and reaction of OH^−^ ions coming from HMTA.^9^ Hence, the electrostatic interactions between the positively charged crystallographic planes with the Zn^2+^ ions are not expected to play a major role in the nucleation of ZnO NWs.

The intermediate pH region is clearly visualized as blue-colored in Fig. 4. As discussed previously, in this region from pH_0_ of ∼7.60 to ∼9.10, the homogeneous growth is favored at the expense of the heterogeneous nucleation due to the very high supersaturation levels. As depicted in Fig. S4,† the supersaturation ratio in this region reaches a maximum value at a pH of ∼8.30, and later it starts decreasing until reaching again supersaturation values <40 at a pH_0_ of ∼9.2.

Consequently, the high-pH region shows that increasing the pH_0_ value from 10.55–10.94 brings the growth of ZnO NWs to decreasing supersaturation levels. It is worth mentioning that, in the high-pH region, the homogeneous nucleation is highly reduced due to the stabilization of Zn^2+^ ions in the form of Zn(NH_3_)^2+^_4_ ions,^52^ which is well correlated with the higher deposited volume of ZnO NWs, as shown in Fig. 2. In this region, a pH decrease through all the growth of ZnO NWs proceeds, contrasting the case in the low-pH region. Hence, this trend supports the deposition mechanism discussed by D. Lincot, where Case 2 deposition process is associated with a pH shift to lower values.^6^

The growth performed in the high-pH region has a pH_0_ value where both the *c*- and *m*-planes are negatively charged; however, after ∼20 min of growth, the pH curves cross the *m*-plane PZC value, leaving the rest of the growth with the *m*-planes positively charged and the *c*-plane negatively charged. From a thermodynamic approach, the growth of ZnO NWs is driven by surface energy minimization through the preferred formation of the non-polar *m*-plane, which has a lower surface energy as compared with the polar *c*-plane.^50^ Hence, the high axial growth rate giving a pencil-like morphology to the ZnO NWs is well-explained by combining this highly reactive crystallization along the *c*-plane^50^ and the electrostatic forces between the positively charged Zn(NH_3_)^2+^_4_ species and the negatively charged *c*-plane. It is worth noting that the ZnO NWs grown at pH_0_ values of 10.55 and 10.62 enter and stay in the *c*-plane PZC region for ∼120 min. This could explain why the axial growth rate of these ZnO NWs is lower than the ZnO NWs grown with pH_0_ > 10.70, and their narrow-hexagonal tip. As shown in Fig. S3,† it is also evident that at a pH_0_ of 11.07, the solubility of Zn(ii) species is so high that the ZnO seed layer is even dissolved. These results correlate and draw attention to the high importance of the type of Zn(ii) species, the supersaturation levels, and the electrostatic interactions occurring within the CBD solution, all of which depending strongly on pH.

### 3.2 Structure of crystal defects using density functional theory

**Fig. 5:**
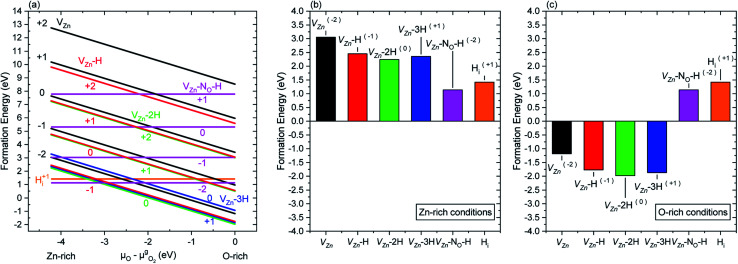
Formation energy of nitrogen- and hydrogen-related defects in ZnO represented (a) as a function of the oxygen chemical potential, (b) under Zn-rich conditions, and (c) under O-rich conditions. The formation energies were obtained from DFT calculations considering that the Fermi level is set to the CBM. For each charge state, only the most stable configuration with the lowest formation energy is represented.

The formation energy of nitrogen- and hydrogen-related defects in ZnO was carefully investigated by DFT calculations following the methodology as reported in ref. 33 and 35, using the VASP code^36,37^ with PAW potentials under the GGA-PBE approximation.^38,39^ In ref. 33 and 35, the *μ*_O_ value was set to −2.11 eV to emulate intermediate growth conditions. In the present investigation, the formation energy of the main nitrogen- and hydrogen-related defects was calculated within the *μ*_O_ range of −4.22 to 0 eV when the Fermi level is set to the conduction band minimum (CBM), as depicted in Fig. 5(a). Two different behaviors related to the evolution of the formation energy of nitrogen- and hydrogen-related defects are revealed: (i) a steady decrease in the formation energy of the V_Zn_, V_Zn_-H, V_Zn_-2H, and V_Zn_-3H defects as *μ*_O_ approaches the O-rich condition and (ii) a constant formation energy of H_BC_ (H_i_) and V_Zn_-N_O_-H defects, regardless of *μ*_O_. Additionally, the formation energy depicted as histograms under the Zn- and O-rich conditions is shown in Fig. 5(b) and (c), respectively. These figures give a better visualization of the two different behaviors described previously. These results are comparable with the results of Fabbri *et al.*^53^

A complex, yet elegant approach was developed by Todorova *et al.*, where the correlation between DFT calculations and experimentally measurable quantities, such as the pH and electrode potential, was associated with the state of an electrochemical system.^27,28^ Using this approach and correlations, we can state that for a given electron potential, an increase in the pH of the CBD solution would promote a reasonable increase in *μ*_O_. Hence, this indicates that by increasing pH_0_ (and consequently *μ*_O_) in the CBD solution, the formation of a higher concentration of V_Zn_-related defects (with the exception of V_Zn_-N_O_-H defects) could be promoted owing to their lower formation energy.

### 3.3 Cathodoluminescence spectroscopy of the as-grown and annealed ZnO nanowires

**Fig. 6:**
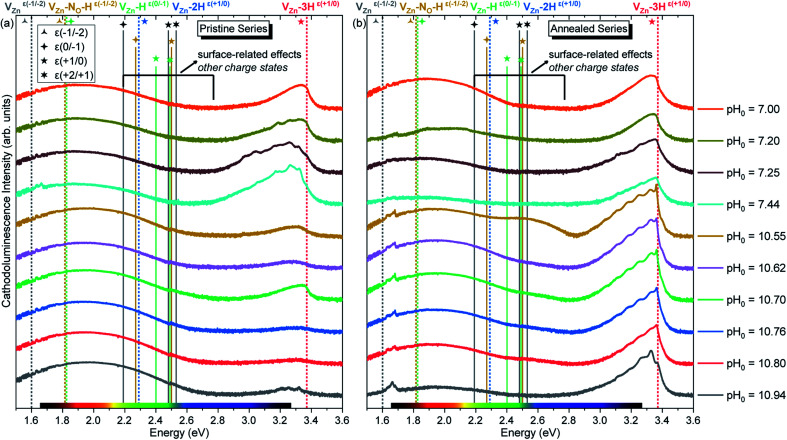
5 K cathodoluminescence spectra of ZnO NWs grown by CBD with pH_0_ values in the range of 7.00–10.94 (a) before and (b) after thermal annealing at 450 °C for 1 h under an oxygen atmosphere. The insets show the emission energy of optical transitions as inferred from DFT calculations in ref. 29, 33, 35 and 61.

The pristine series of ZnO NWs grown by CBD using pH_0_ values in the range of 7.00–10.94 was thermally annealed at 450 °C under an oxygen atmosphere, following the methodology developed in ref. 35. This additional set of ZnO NWs for our study is defined as the annealed series. The 5 K cathodoluminescence spectra for the pristine and annealed series of ZnO NWs grown by CBD are presented in Fig. 6. The near band edge (NBE) emission for the pristine series shows a strong difference between the ZnO NWs grown in the low-pH and high-pH regions, as revealed in Fig. 6(a). In the low-pH region, ZnO NWs grown under standard conditions using the pH_0_ value of 7.00 show a NBE emission with predominant radiative transitions involving neutral donor-bound A-excitons (D°X_A_) at ∼3.365 eV. The contributions from H_O_ (I_4_), V_Zn_-3H defect complex (I_5_) and H_BC_ lines at 3.3628, 3.3614, and 3.360 eV, respectively, are expected in that NBE emission.^33,54–56^ Short ZnO NWs grown with the pH_0_ value of 7.44 (see Fig. 1) show two prominent emission lines at 3.3288 eV and 3.330 eV (*i.e.* Y_0_ line). They are respectively attributed to the basal plane stacking faults^57^ and extended defects^58^ presumably located at their bottom, where a compact layer is formed following a coalescence process as in the case of polycrystalline ZnO thin films.^59^ In contrast, ZnO NWs grown in the high-pH region show a moderately suppressed NBE emission possibly due to competition with the radiative recombination of defects in the visible spectral region, or the presence of non-radiative recombination centers, such as dislocations, point defects and surface/interface states.^60^

**Fig. 7:**
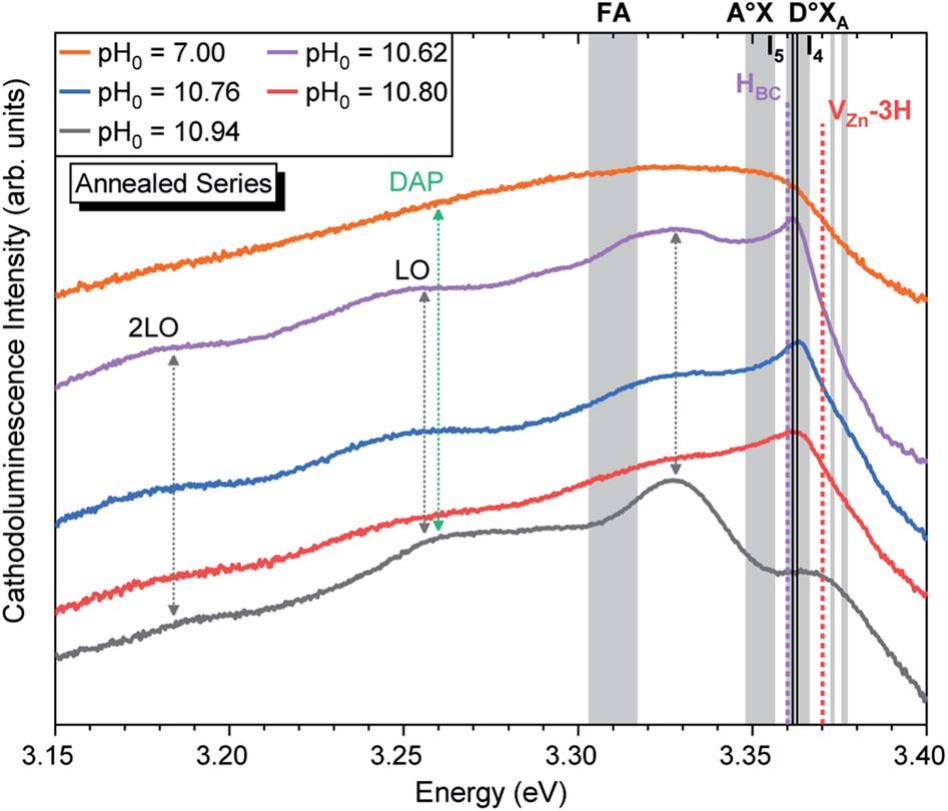
5 K cathodoluminescence spectra located in the NBE emission region of ZnO NWs grown by CBD using pH_0_ values in the range of 7.00–10.94 after thermal annealing at 450 °C for 1 h under an oxygen atmosphere. The insets show the emission energy of optical transitions as inferred from DFT calculations in ref. 33, 54 and 56.

As suggested by Wang *et al.*, a high density of vacancies in the surface region is detrimental for the NBE emission.^60^ This is well-correlated with the expected high density of V_Zn_-related defects as suggested by our DFT calculations. Following this hypothesis, a reduction of these V_Zn_-related defects would greatly enhance the NBE emission, and effectively this is what is seen in Fig. 6(b) and 7. After performing thermal annealing at 450 °C under an oxygen atmosphere, the NBE emission intensity of the annealed ZnO NWs is strongly increased and the contribution of additional emission lines occurs. As a comparison, the annealed ZnO NWs grown at a pH_0_ value of 7.00 is centered at ∼3.329 eV. The widening of the NBE under similar conditions has been shown due to the contribution of radiative transitions involving neutral acceptor-bound A excitons (A°X_A_) arising from the activation of V_Zn_-N_O_-H defect complexes at ∼450 °C.^35^

Fig. 7 shows that the annealed ZnO NWs grown in the high-pH region exhibit an interplay between the D°X_A_ transitions and new emission lines with prominent shoulders related to nitrogen-doped ZnO around the A°X_A_,^62,63^ donor–acceptor pair (DAP),^64–66^ free electron-to-acceptor (FA)^67^ transitions, and LO phonon replicas separated by a phonon energy of 72 meV.^55^ These emissions have already been seen in 800–900 °C annealed ZnO NWs grown by CBD under standard conditions (*i.e.* pH_0_ of ∼7.00).^35^ In annealed ZnO NWs grown using the pH_0_ values ranging from 10.55 to 10.76, the NBE emission is dominated by a sharp line arising at ∼3.360 eV, which is in good correlation with the I_4_, I_5_, and H_BC_ lines.^33,54–56^ These are also accompanied by a prominent shoulder located at ∼3.33 eV, within the energy range of the two-electron satellite (TES) transitions originating from the corresponding I_4_ line.^55^ Then, the annealed ZnO NWs grown using the pH_0_ value of 10.94 show a NBE emission centered at 3.327 eV. This important red-shift from the D°X_A_ transitions indicates the involvement of emission lines attributed to DAP, and FA transitions with the contributions from the V_Zn_, V_Zn_-H and V_Zn_-N_O_-H defects acting as deep acceptors in the bulk of ZnO NWs,^33,35,68^ suggesting a possible acceptor–donor compensation process.

The broad emissions in the visible spectral region are attributed to (i) the red–orange band centered at ∼1.85 eV, (ii) the yellow–green band centered at ∼2.30 eV, and (iii) the green–blue band centered at ∼2.66 eV, as shown in Fig. 6. The red–orange emission band is attributed to the (0,−1) and (−1,−2) transition levels of the V_Zn_-H and V_Zn_-N_O_-H defect complexes acting as deep acceptors, respectively, and both of them share a theoretical emission energy of 1.82 eV.^33,35^ The yellow–green emission band is attributed to the neutral V_Zn_-2H defect complex, and additional contributions at ∼2.27 and 2.40 eV from the (0/−1) and (+1/0) transition levels of the V_Zn_-N_O_-H and V_Zn_-H defects located on the surfaces of ZnO NWs could be seen.^35^ The green–blue emission band is labeled as related to surface effects, since it is attributed to unexpected charge states coming from the recombination of electrons with the high density of holes that migrate to the ZnO NW surfaces due to the upward band-bending caused by the adsorbed oxygen ions.^33,69^ The emissions falling in the surface-effect related region involve the nitrogen- and hydrogen-related defects including V_Zn_-H,^33^ V_Zn_-2H,^33^ and V_Zn_-N_O_-H defect complexes.^35^

**Fig. 8:**
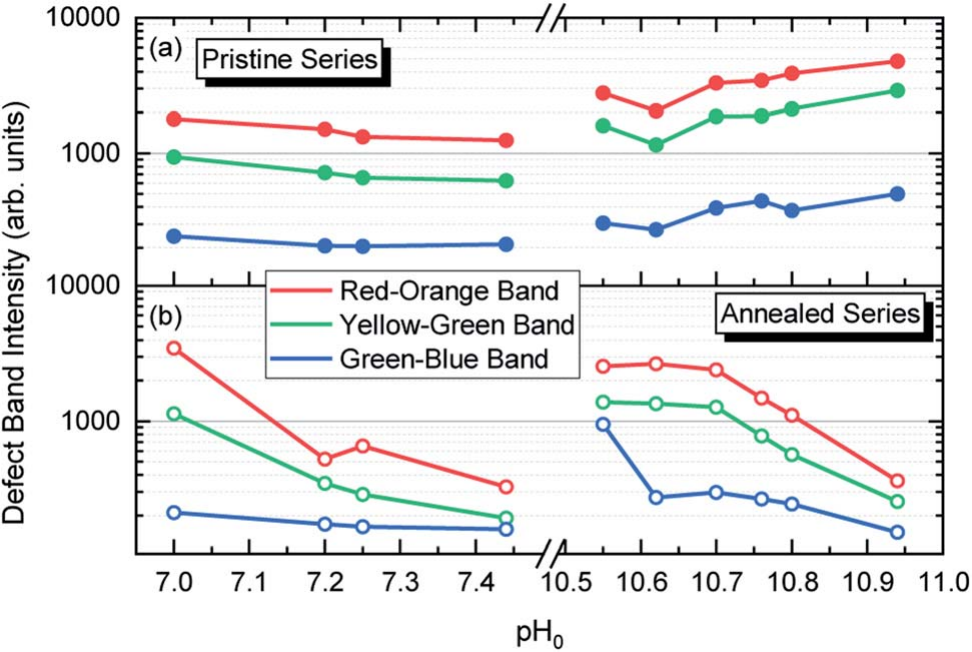
Evolution of the relative intensity of the cathodoluminescence emission bands located in the visible spectral region of ZnO NWs grown by CBD using pH_0_ values in the range of 7.00–10.94: (a) before and (b) after annealing at 450 °C for 1 h under an oxygen atmosphere.

The intensity of the visible emission bands for both the pristine and annealed series of ZnO NWs is presented in Fig. 8. The pristine series of ZnO NWs shows an intensity that follows a similar trend between the red–orange, yellow–green, and green–blue emission bands for both the low-pH and high-pH regions: the intensity of the defect bands in ZnO NWs grown in the low-pH region continuously decreases as the pH_0_ value is increased from 7.00 to 7.44, while the same intensity in ZnO NWs grown in the high-pH region follows an upward trend as the pH_0_ value is increased from 10.55 to 10.94. The annealed series of ZnO NWs also show a decreasing intensity of the red–orange emission band along the low-pH region from 7.00–7.44. At a pH_0_ value of 7.00, the intensity of the red–orange emission band is 3× and 16× higher than the yellow–green and green–blue emission bands, respectively. While increasing the pH_0_ value from 7.00 to 7.44, a strong decrease of ∼90% is observed for the three defect bands in ZnO NWs. Additionally, a clear increase in the intensity of the red–orange emission band from the pristine series to the annealed series is seen in ZnO NWs grown with the pH_0_ value of 7.00, which could again indicate the preferential formation of both V_Zn_-H and V_Zn_-N_O_-H defect complexes after thermal annealing at 450 °C.^35^ The increase in the concentration of V_Zn_ during the thermal annealing under an oxygen atmosphere is favorable for the preferential formation of low-coordinated V_Zn_-*n*H defect complexes.^70^ Interestingly, a simultaneous strong increase in the intensity of the three defect emission bands is revealed in ZnO NWs grown with the pH_0_ value of 10.55. The strong red–orange and yellow–green emission bands indicate the formation of the V_Zn_-H and V_Zn_-2H defect complexes in the bulk of ZnO NWs as well as of the V_Zn_, V_Zn_-H and V_Zn_-N_O_-H defects with unexpected charge states on their surfaces, which is very well correlated with the NBE widening towards the A°X_A_, DAP, and FA emissions. The increase in the pH_0_ value to 10.70 maintains a maximum intensity of the red–orange and yellow–green emission bands in ZnO NWs, and then the further increase in the pH_0_ value to 10.94 slowly decreases the intensity of the defect emission bands. Hence, the intense red–orange and yellow–green emission bands in the high-pH region reveal the massive incorporation of (i) V_Zn_-related defects favored by their low formation energy as shown in Fig. 5, and (ii) nitrogen-related defects favored by the attractive electrostatic interactions as presented in Fig. 4.

### 3.4 Raman spectroscopy of the as-grown and annealed ZnO nanowires

**Fig. 9:**
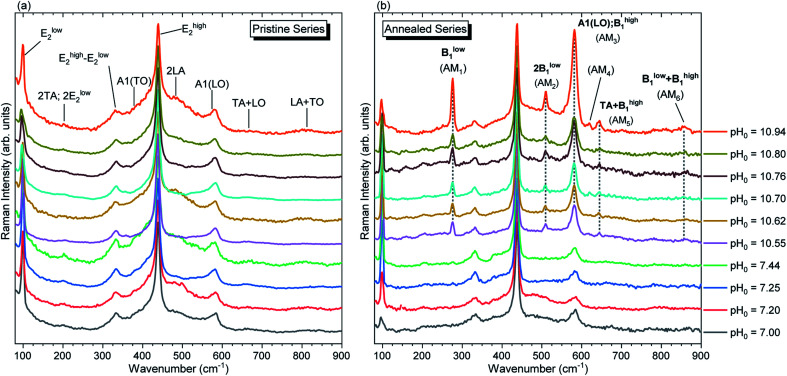
Raman scattering spectra focused on the low wavenumber region of ZnO NWs grown by CBD using pH_0_ values in the range of 7.00–10.94: (a) before and (b) after thermal annealing at 450 °C for 1 h under an oxygen atmosphere. The inset labels show the assigned vibrational frequencies following ref. 71 and 73.

The Raman scattering spectra collected in the low wavenumber region for both pristine and annealed series of ZnO NWs are presented in Fig. 9. In the low wavenumber range of 50–900 cm^−1^, the characteristic optical phonon modes for the wurtzite structure of ZnO NWs are observed at 99 (E^low^_2_), 378 (A_1_(TO)), 438 (E^high^_2_), and 574 (A_1_(LO)) cm^−1^.^71^ The Raman lines at 203 (2TA/2E^low^_2_), 333 (E^high^_2_ − E^low^_2_), 483 (2LA), 666 (TA + LO), and 812 (LA + TO) cm^−1^ correspond to the second-order modes. These modes are evident for both the pristine and annealed series of ZnO NWs in Fig. 9(a) and (b), respectively. Fig. 9(b) shows the appearance of additional modes (AMs) after thermal annealing on the ZnO NWs grown in the high-pH region, which are summarized in Table 2. These AMs have also been reported in ZnO NWs grown by CBD, where the intentional doping with Al and Ga has been achieved.^13,15^ AMs are systematically related to the presence of extrinsic impurities including Al, Ga, Sb, and Fe^72^ in ZnO, and are attributed to dopant-induced defects in the host lattice and/or to ZnO B_1_ silent modes that become Raman-active.^73,74^

**Table 2:** Frequency of additional Raman modes measured in annealed ZnO NWs grown by CBD using pH_0_ values in the range of 10.55–10.94

	*ω*_*Ab initio*_ a (cm^−1^)	Mode definition^a^	*ω*_exp_ b (cm^−1^)	Assigned mode^b^
AM_1_	261	B^low^_1_	276	B^low^_1_
AM_2_	520	2B^low^_1_	509	2B^low^_1_
AM_3_	552	B^high^_1_	581	A_1_(LO); B^high^_1_
AM_5_	650	TA + B^high^_1_	644	TA + B^high^_1_
AM_6_	810	B^low^_1_ + B^high^_1_	856	B^low^_1_ + B^high^_1_

a Ref. 73.

b Experimental data.

In principles, the influence of the chemical nature of a foreign atom or the vacancy of a native atom within the ZnO host gives a change of the electronic potential in the neighboring atoms,^75,76^ which directly influences the electronic charge density, structural parameters, atomic displacements and phonon frequencies in the crystal structure.^73,76,77^ Since ZnO exhibits a large orbital hybridization,^78^ the point defects affect the atomic vibrational interactions related to the short-range interactions with the nearest neighbors (NNs) and next-nearest neighbors (NNNs), which are highly related to the screening effect due to the bonding electrons, following the rigid-ion model for lattice-dynamic calculations.^76^ This could account for the appearance of AMs after thermal annealing at 450 °C under an oxygen atmosphere *via* (i) the structural relaxation, reduction of free charge carriers and activation of nitrogen-related defects,^35^ and (ii) the reduction of V_Zn_-related defects, as suggested by the NBE enhancement discussed previously. These two effects seem to play a role in the reduction of the static and dynamic charges,^79,80^ enhancing the vibrational interactions, which are consequently manifested as AMs in the Raman spectra of annealed ZnO NWs grown in the high-pH region.

Manjón *et al.* reviewed relevant literature of undoped and doped ZnO thin films and compared it with *ab initio* calculations in ref. 73. In this manner, it was shown that these AMs correspond to B_1_ silent modes from the wurtzite structure of ZnO, which are observed by disorder-activated Raman scattering due to the breakdown of the translational symmetry of the crystal lattice.^74^ Following these assignments, the experimental position of the Raman lines related to AMs can be correlated with the expected position of the B_1_ modes as detailed in Table 2. The Raman lines at 276 cm^−1^ (AM_1_) and 581 cm^−1^ (AM_3_) correspond to the B^low^_1_ and B^high^_1_ modes. Since the B_1_ modes share the same scattering behavior as the E_2_ modes, their FWHM would be greatly influenced by the two-phonon density of states (DOS).^81^ Indeed, Fig. S5(a)† shows that B^low^_1_ located in a low DOS region and B^high^_1_ located in a high DOS region have an average FWHM of ∼9.5 cm^−1^ and ∼16.3 cm^−1^, respectively. It is important to note that the B^high^_1_ mode falls near the 574 (A_1_(LO)) cm^−1^ mode and a proper decoupling between the two modes is not possible due to their proximity. The 
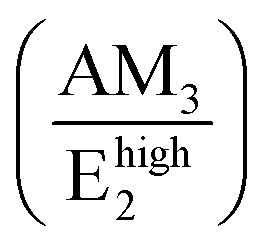
 relative intensities of the annealed ZnO NWs grown in the low- and high-pH regions are ∼0.12 and ∼0.32, respectively. The higher relative intensity in the high-pH region (2.7×) could easily be explained by the additional contribution of the B^high^_1_ mode. For example, the annealed ZnO NWs grown at a pH_0_ of 10.94 has the highest 
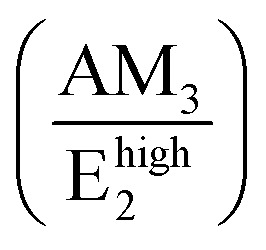
 relative intensity of 1.08 (Fig. S5(b)†). Interestingly, we can also see the appearance of AM_4_ located at 619 cm^−1^, with a fairly weak intensity which has been attributed to iron, aluminium, and gallium dopants in ref. 72 and it was later assigned to the second-order TA + TO mode in ref. 71. Lattice distortions can enhance the TA and TO modes due to their dependence on the Zn-like and/or O-like vibrations, which are correlated with the high density of one-phonon and two-phonon DOSs along these wavelengths.^73^ The presence of B^low^_1_, B^high^_1_, and AM_4_ along with the observed high intensity of AM_3_ helps to assign second-order modes such as the modes seen at 644 cm^−1^ (AM_5_) and 856 cm^−1^ (AM_6_) corresponding to TA + B^high^_1_ and B^low^_1_ + B^high^_1_, respectively.^74^ Additionally, the LO phonon replicas observed in Fig. 6 have recently been correlated with the activation of the B^high^_1_ mode due to the strong coupling between the A_1_(LO), B^high^_1_ polar phonons (∼580 cm^−1^) and the photo-generated electrons.^82^

**Fig. 10:**
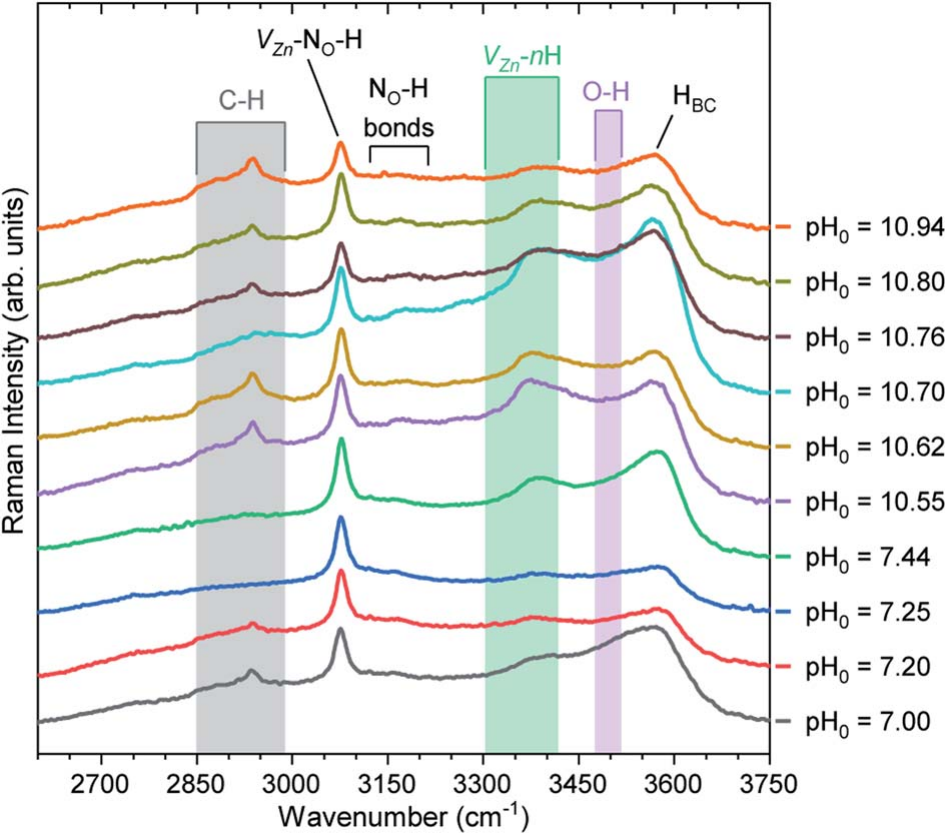
Raman scattering spectra collected in the high wavenumber region of pristine ZnO NWs grown by CBD using pH_0_ values in the range of 7.00–10.94. The inset labels show the assigned vibrational frequencies following ref. 29, 35, 68, 83, 86, 87, 88 and 89.

Fig. 10 shows the Raman scattering spectra collected in the high wavenumber range of 2600–3750 cm^−1^ for the pristine series of ZnO NWs. This region is crucial to visualize the Raman lines associated with the presence of carbon-, nitrogen-, and hydrogen-related defects. In the 2750–3000 cm^−1^ region, we can see C–H_*X*_ groups (*X* = 1, 2, 3)^83^ coming from the surfaces of ZnO NWs. It has been shown that residual HMTA molecules are adsorbed on the nonpolar *m*-planes, inducing the occurrence of the C–H_*X*_ groups.^8,9,84^ These C–H_*X*_ modes can be seen in all ZnO NWs, with the exception of the ZnO NWs grown at the pH_0_ values of 7.25 and 7.44. In fact, as shown in Fig. 2, the ZnO NWs grown at pH_0_ values of 7.25 and 7.44 possess the two lowest aspect ratios, hence the related minimal surface area makes it reasonable for the C–H_*X*_ mode intensity to be weaker.

A prominent Raman line centered at 3078 cm^−1^ is attributed to the V_Zn_-N_O_-H defect complex acting as a deep acceptor presumably located on the surfaces and in the bulk of ZnO NWs.^35,68^ We can clearly observe that the Raman line assigned to the V_Zn_-N_O_-H defect complex is fairly intense, regardless of the pH_0_ value. Smaller in intensity, the Raman lines located at ∼3121 and ∼3160 cm^−1^ separated by around 40 cm^−1^ are attributed to the N_O_-H bonds in the AB_N⊥_ and AB_N‖_ configurations.^35,85^ It has been suggested that these nitrogen-related defects can be located in the center or on the surfaces of ZnO NWs,^35^ and originate from residual nitrogen coming from HMTA molecules.^9^

It is well-known that the Raman line at 3575 cm^−1^ is assigned to H_BC_ acting as a shallow donor,^86,87^ and it generally has an asymmetric shape due to the weak shoulder around 3500 cm^−1^ attributed to the O–H bonds^68,86,88^ located on the surfaces of ZnO NWs. Additionally, the contribution of V_Zn_-*n*H defect complexes in the 3300–3418 cm^−1^ region is due to the V_Zn_-H,^29,86^ V_Zn_-2H,^86^ and V_Zn_-3H defects.^89^ The Raman line related to H_BC_ is generally the most prominent Raman line assigned to hydrogen-related defects in ZnO NWs grown by CBD under standard growth conditions, as given by the pH_0_ value of 7.00. However, the increase in the pH_0_ value used for growing ZnO NWs by CBD helps to modulate the ratio between the intensities of the Raman lines assigned to H_BC_ and V_Zn_-*n*H defect complexes. In particular in the high-pH region, we can observe a prominent valley forming around the O–H bond band due to the increase in the intensity of the Raman lines assigned to V_Zn_-*n*H defect complexes. The prominent V_Zn_-*n*H band is centered at ∼3369 cm^−1^, which is in good correlation with the calculated (∼3361 cm^−1^) and experimental (∼3358 cm^−1^) positions of the V_Zn_-H defect complex acting as a deep acceptor.^29^ This further validates the expected behavior that at higher pH_0_, *μ*_O_ increases and consequently promotes the preferential formation of V_Zn_-*n*H defect complexes due to its reduced formation energy. In our particular case, the V_Zn_-*n*H defect complexes seem to massively form with the lower coordinated V_Zn_-H defect complex, which correlates well the diminished NBE emission seen for the pristine series of ZnO NWs grown in the high-pH region.

By studying the E^high^_2_ and E^low^_2_ modes, we can gain insight into the effects of the intrinsic/extrinsic point defects inside the crystal lattice of the ZnO NWs. The E^high^_2_ mode is mainly related to the motion of the O lattice and is known to shift toward higher wavenumber values when the ZnO lattice is in a compressive stress state.^71,80^ The E^high^_2_ mode of the pristine ZnO NWs is centered at ∼438.6 cm^−1^ and has a FWHM of ∼9.0 cm^−1^, and, after thermal annealing, the mode shifts to ∼437.5 cm^−1^ and has a FWHM of ∼7.3 cm^−1^, as shown in Fig. S6.† This ∼1.1 cm^−1^ shift in all the annealed ZnO NWs suggests a moderate relaxation due to thermal annealing, where an exodiffusion, redistribution of hydrogen-related defects, and activation of the V_Zn_-N_O_-H defect complex are expected to occur under the present conditions.^35^ The pristine series shows a systematic increase of the E^high^_2_ mode position from 438.3 to 438.9 cm^−1^, suggesting the increase in the strain as the pH_0_ value increases in the high-pH region. The annealed series shows an interesting systematic decrease from the pH_0_ of 7.00 up to 10.70 where it reaches the minimum position of 437.1 cm^−1^, and then the further increase in the pH_0_ value gives a slight increase of the position up to 437.5 cm^−1^. Studies on pressure-induced effects on ZnO modes in ref. 80 and 90 suggest that this relaxation is related to the weakening of the Born transverse dynamic charge within the crystal structure. In contrast, the E^low^_2_ mode shown in Fig. S7† is mainly related to the motion of Zn lattice.^71^ Its position shows no evident difference between the pristine and annealed series, and also no evident trend is detected between the low-pH and high-pH regions. Furthermore, in Fig. S8,† we further analyze the intensity ratio of E^low^_2_ and E^high^_2_ and only the established difference between the pristine and annealed series is seen. With the previous analysis in the E^high^_2_ and E^low^_2_ modes, we can expect that the enhanced vibrational interactions are due to the observed trend on the motion derived from the O lattice.

*Via* Raman spectroscopy, the presence of V_Zn_-, carbon- and nitrogen-related defects is revealed from the presence of their related vibrational modes. In the present study, the incorporation of extrinsic point defects would be attributed to the impurities in the chemical precursors used and the nature of the species present in solution. The atomic weight (%) relative to zinc atoms of foreign elements present in the CBD reaction at pH_0_ values of 7.00 and of 10.94 is presented in Fig. S9(a) and (b),† respectively. In both cases, nitrogen and carbon are highly present in the chemical bath since their atomic weight (%) relative to zinc atoms reaches 128.5 and 110.2%, respectively. As previously discussed, nitrogen comes from both the nitrates and nitrogen atoms present in our precursors. Additionally, in the high-pH region, the dominant amine complexes, *e.g.* Zn(NH_3_)^2+^_4_, could greatly favor nitrogen incorporation. In contrast, carbon is a residual impurity from the methylene bridges coming from the HMTA molecule. Moreover, other residual impurities such as Na, Cl, Fe, Cu, Pb, Cd, Co, Ni and heavy metals are present as well in the bath, however in a much lower ratio ranging from 0.2 to 5% atomic weight.

### 3.5 Effects of pH on the CBD solution and overall properties of ZnO NWs

**Fig. 11:**
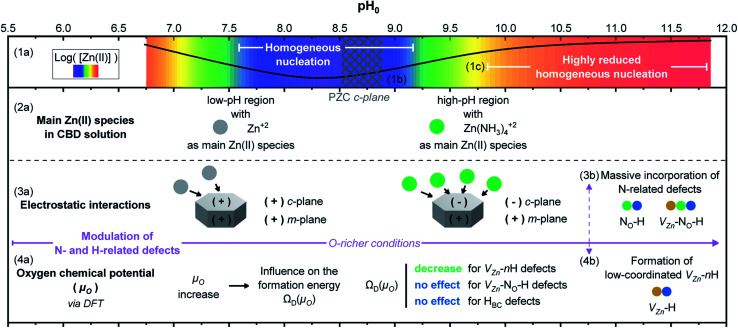
Schematic illustration showing an overall summary of the influence of pH_0_ on the CBD solution for the growth of ZnO NWs. (1a) The color-coded map illustrates the theoretical equilibrium Zn(ii) species concentration as calculated using Visual MINTEQ with a black solid line and (1b) the PZC of the polar *c*-plane is represented by a meshed area. (1c) One region shows where the homogeneous nucleation is highly promoted and other region where it is highly reduced. (2a) The main Zn(ii) species in the CBD solution in the low- and high-pH region are depicted. (3a) The electrostatic interactions existing between the ZnO NW surfaces and the charged Zn(ii) species in solution illustrate how a massive incorporation of nitrogen-related defects is expected in the high-pH region (3b). (4a) The relation between the pH of the CBD solution, the oxygen chemical potential (*μ*_O_) and the formation energy of point defects *Ω*_D_(*μ*_O_) is shown. (4b) Due to the modulation of *μ*_O_, a massive formation of low-coordinated V_Zn_-*n*H defects is revealed. Note: the Zn^2+^ and Zn(NH_3_)_4_^2+^ions, as well as the N_O_-H, V_Zn_-N_O_-H, and V_Zn_-H defects are represented *via* spheres for illustration purposes with no real scale.

The variation of pH_0_ in the CBD solution has great consequences on the overall properties of ZnO NWs, as is summarized in Fig. 11. The schematic illustration is meant to be read from top to down. In the first frame, the theoretical equilibrium concentration of Zn(ii) species (1a) is depicted combining the color-coded map and plot from Fig. 4 and S3(b)† at room temperature. The two favorable regions for the heterogeneous nucleation and growth of ZnO NWs going from green to orange–red sections, namely the low-pH and high-pH regions, are clearly delineated. In contrast, the blue–purple section represents the region with high supersaturation levels, where homogeneous nucleation and growth are favored.

In the second frame, the main Zn(ii) species in the CBD solution (2a) are obtained from thermodynamical calculations using Visual MINTEQ, where Zn^2+^ ions are the main species in the low-pH region and then Zn(NH_3_)_4_^2+^ ions are the main species in the high-pH region, as detailed in Fig. 3. The stabilization of the Zn(ii) species as amine complexes allows an important reduction of the homogeneous nucleation (1c) in the high-pH region,^52^ which clearly explains the higher deposited volume, as shown in Fig. 2(e). Given that the elongation of ZnO NWs is thermally activated at ∼80 °C,^10^ the growth of NWs operates under the conditions where the low-pH region shows both the *c*-plane and *m*-planes of the NW positively charged, whereas the high pH region has the *c*-plane negatively charged and the *m*-plane positively charged. This surface charge change of the polar *c*-plane is shown by its PZC (1b). These conditions help to gain great understanding of the formation mechanisms, while taking into account the electrostatic interactions (3a) between the Zn(ii) species and the charged surfaces of the crystallographic planes of ZnO NWs. As shown in Fig. 1 and 2, it was evident that ZnO NWs grown in the high-pH region have a considerable higher axial growth rate (>1 μm h^−1^) and aspect ratio (>20) that give the NWs a narrower pencil-like tip, as compared with ZnO NWs grown in the low-pH region. Fig. 11 helps to illustrate the strong electrostatic interactions we could expect between the negatively charged *c*-plane and the positively charged Zn(NH_3_)_4_^2+^ ions, hence explaining the faster nucleation on the *c*-plane growth front giving the narrow tips on the NWs grown with a pH_0_ value of 10.55–10.94.

Following the work by Todorova *et al.*, the formation of point defects in ZnO NWs is also greatly influenced by the pH of the CBD solution,^27,28^ and it was further investigated in this study *via* DFT calculations. Increasing the pH_0_ of the solution brings the chemical environment to oxygen-richer conditions (*i.e.* increasing oxygen chemical potential (*μ*_O_)) (4a), having a direct effect on the formation energy *Ω*_D_(*μ*_O_) of point defects. In Fig. 5, the formation energy of V_Zn_, V_Zn_-H, V_Zn_-2H, and V_Zn_-3H defects steadily decreases as *μ*_O_ shifts to O-rich conditions, while the formation energy of H_BC_ and V_Zn_-N_O_-H defects is independent upon this shift. This was greatly evidenced in Fig. 6(a) where a diminished NBE emission is observed on the pristine ZnO NWs grown in the high-pH region due to the great incorporation of V_Zn_-related defects located in the visible spectral region.^33,35^ And it is also evident in Fig. 10 where the V_Zn_-*n*H mode centered at ∼3369 cm^−1^ becomes more prominent than the H_BC_ and V_Zn_-N_O_-H modes in the high-pH region. Hence, this confirms the massive and favorable incorporation of low coordinated V_Zn_-*n*H defects due to the lower formation energy as *μ*_O_ is increased (4b).

DFT calculations in Fig. 5 showed that the incorporation of V_Zn_-N_O_-H defect complexes is not expected to be higher by increasing *μ*_O_. However, given the fact that the Zn(NH_3_)_4_^2+^ ions are the most stable Zn(ii) species in the high-pH region and that these positively charged ions have a strong electrostatic interaction with the negatively charged polar *c*-plane, we could expect an important incorporation of nitrogen along the crystallization of the ZnO NWs grown in this region (3b). It has been suggested that impurities substituting for an oxygen site would give higher perturbation on the lattice as compared to impurities substituting for a Zn site.^74^ Under the present conditions, the high incorporation of V_Zn_-related, V_Zn_-N_O_-H and N_O_-H defects in the high-pH region may explain the AMs related to the disorder-activated Raman B_1_ silent modes as observed in Fig. 9. However, while the lattice distortion is attributed to V_Zn_- and N_O_-related defects, an important incorporation of other dopants, such as Fe, Sb, Al, and Ga could also promote a distortion of the lattice and the appearance of these additional B_1_ silent modes, as suggested in ref. 13, 15, 19 and 72.

## 4. Conclusion

In summary, we have shown that the pH_0_ adjustment of the CBD solution greatly impacts the formation of nitrogen- and hydrogen-related defects in ZnO NWs, allowing their modulation through the electrostatic interactions occurring along the NW nucleation and elongation, and the influence of *μ*_O_ on the formation energy of point defects. Increasing the pH_0_ of the CBD solution strongly influences the main species present in the bath, where in the high-pH region the stabilized Zn(NH_3_)_4_^2+^ ions promote a massive incorporation of nitrogen-related defects due to the strong electrostatic interactions between the positively charged Zn(NH_3_)_4_^2+^ ions and the negatively charged polar *c*-planes. Additionally, increasing the pH_0_ brings the growth of ZnO NWs to O-richer conditions (increased *μ*_O_), which according to our DFT calculations promotes a decrease in the formation energy of V_Zn_ and V_Zn_-*n*H defects, while the formation energy of H_BC_ and V_Zn_-N_O_-H remains invariant. Hence, ZnO NWs grown in the high-pH region are expected to massively form V_Zn_ and V_Zn_-*n*H defects and this is clearly observed with (i) the prominent Raman line at ∼3369 cm^−1^ attributed to the low coordinated V_Zn_-H defect complex, (ii) the strong emission bands in the red–orange and yellow-green spectral regions mainly attributed to the V_Zn_-H, V_Zn_-N_O_-H and V_Zn_-2H defect complexes, and (iii) the diminished NBE emission for the pristine series, which is later enhanced through thermal annealing from the reduction of these V_Zn_-related defects. Interestingly enough, the massive incorporation of V_Zn_- and nitrogen-related defects also contributes besides other residual impurities to the disorder-activated Raman B_1_ silent modes which appear after thermal annealing at 450 °C under an oxygen atmosphere due to the strong distortion and relaxation of the crystal structure. The modulation of the nitrogen- and hydrogen-related defects in unintentionally doped ZnO NWs grown by CBD with a controlled pH_0_ value brings us one step closer towards their more efficient application in nanoscale engineered devices.

## Conflicts of interest

There are no conflicts of interest to declare.

## Supplementary Material

NA-004-D1NA00785H-s001
