# Supplementary material for: Modulating the growth of chemically deposited ZnO nanowires and the formation of nitrogen- and hydrogen-related defects using pH adjustment
Source: Nanoscale Adv. 2022 Feb 23;4(7):1793–807. doi: 10.1039/d1na00785h (PMC9417859; doi:10.1039/d1na00785h)
Supplement: NA-004-D1NA00785H-s001 [file NA-004-D1NA00785H-s001.pdf]

Electronic Supplementary Material (ESI) for Nanoscale Advances.
This journal is © The Royal Society of Chemistry 2022

ELECTRONIC SUPPLEMENTARY INFORMATION

Modulating the growth of chemically deposited ZnO nanowires and the formation of nitrogen- and hydrogen-related defects using the pH adjustment

José Villafuerte,[1,2] Eirini Sarigiannidou,[1] Fabrice Donatini,[2] Joseph Kioseoglou,[3] Odette Chaix-Pluchery,[1] Julien Pernot,[2] and Vincent Consonni[1*]

[1] *Univ. Grenoble Alpes, CNRS, Grenoble INP, LMGP, F-38000 Grenoble, France*

[2] *Univ. Grenoble Alpes, CNRS, Grenoble INP, Institut NEEL, F-38000 Grenoble, France*

[3] *Physics Department, Aristotle University of Thessaloniki, 54124 Thessaloniki, Greece*

*Corresponding Authors: julien.pernot@neel.cnrs.fr and vincent.consonni@grenoble-inp.fr

Section S1. Density-Functional Theory Method

The formation energy of defects requires examining the modification of the chemical potentials of species upon introducing a vacancy or impurity atoms in the simulation supercell. In pure bulk ZnO, the chemical potentials should satisfy the following relations [1]:

$$\mu^{s}_{ZnO} = \mu_{Zn} + \mu_{O}$$

$$\Delta H^{f}_{ZnO} = \mu^{s}_{ZnO} - \mu^{s}_{Zn} - \mu^{g}_{O_2}$$

where (ΔH^{f}_{ZnO}) is the heat of formation of ZnO, (μ^{s}_{ZnO}) is the chemical potential of the bulk ZnO in the wurtzite phase and, (μ^{s}_{Zn}) and $(\mu^{g}_{O_2})$ are the chemical potentials of the condensed (Zn metal) and of the gaseous (O_2) phases, respectively. Then these expressions can conveniently be re-written as:

$$\mu_{Zn} = \mu^{s}_{Zn} + (1 - \lambda) \times \Delta H^{f}_{ZnO}$$

$$\mu_{O} = \mu^{g}_{O_2} + \lambda \times \Delta H^{f}_{ZnO}$$

Where $\lambda\ (0 \le \lambda \le 1)$ is a parameter defining O-rich $(\lambda = 0)$ or Zn-rich $(\lambda = 1)$ conditions. The chemical potential difference between species is then given by:

$$\Delta\mu = \left(\mu_{Zn} - \mu^{s}_{Zn}\right) - \left(\mu_{O} - \mu^{g}_{O_2}\right) = \left(\mu_{Zn} - \mu_{O}\right) - \left(\mu^{s}_{Zn} - \mu^{g}_{O_2}\right) = (1 - 2\lambda) \times \Delta H^{f}_{ZnO}$$

And should satisfy the obvious condition:

$$\underset{\substack{(\lambda = 1)\\ Zn - rich}}{-\Delta H^{f}_{ZnO}} \le \Delta\mu \le \underset{\substack{(\lambda = 0)\\ O - rich}}{\Delta H^{f}_{ZnO}}$$

The formation energy of an electrically neutral defect at $T = 0\ K$ is given by [2]:

$$\Omega_D\left(\mu_{Zn},\mu_{O}\right) = E_D - \frac{1}{2}\left(n_{Zn} + n_{O}\right)\mu^{s}_{ZnO} - \frac{1}{2}\left(n_{Zn} - n_{O}\right)\left(\mu^{s}_{Zn} - \mu^{g}_{O_2}\right) - \frac{1}{2}\left(n_{Zn} - n_{O}\right)\Delta\mu$$

Where n_{Zn} and n_{O}, represent the numbers of Zn and O atoms, respectively. By combining the relations above, one easily obtains:

$$\Omega_D\left(\mu_{Zn},\mu_{O}\right) = E_D - E^{total}_{ref} - n'_{Zn}\mu^{s}_{ZnO} - \left(n'_{O} - n'_{Zn}\right)\mu_{O}$$

where, E^{total}_{ref} is the total energy of the reference structure, and n'_{Zn}, n'_{O} are the differences between the numbers of Zn and O atoms in the defected supercells and in the reference structures, respectively. Equally, the formation energy of a defect in a specific charge state (Q) is given by:

$$\Omega_D(\mu_{Zn},\mu_O) = E_D - E^{total}_{ref} - n'_{Zn}\mu^{s}_{ZnO} - (n'_O - n'_{Zn})\mu_O + Q(E^{Fermi}_{D} + E^{VBM}_{ref})$$

where, E^{Fermi}_{D} is the Fermi level energy of the defective system with respect to the Valence Band Maximum (VBM), E^{VBM}_{ref}, in the reference system, shifted by the difference between average electrostatic potentials in the defected and the reference systems.

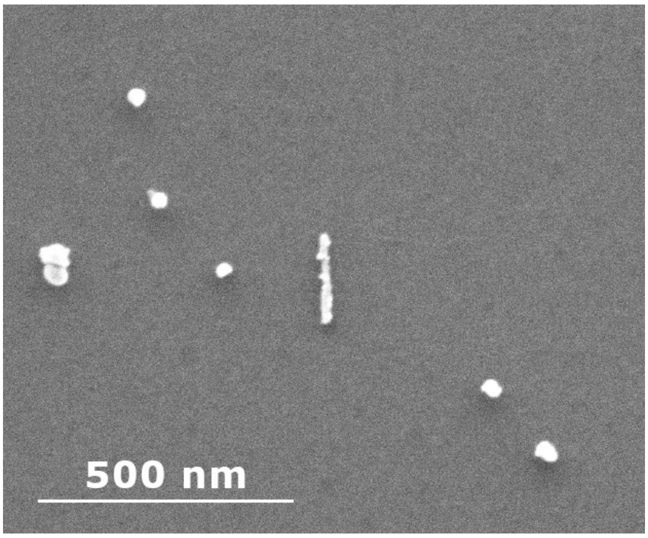

Fig. S2 Top-view field-emission scanning electron microscopy (FESEM) image of the chemical bath deposition (CBD) sample deposited with the pH_0 value of 11.07.

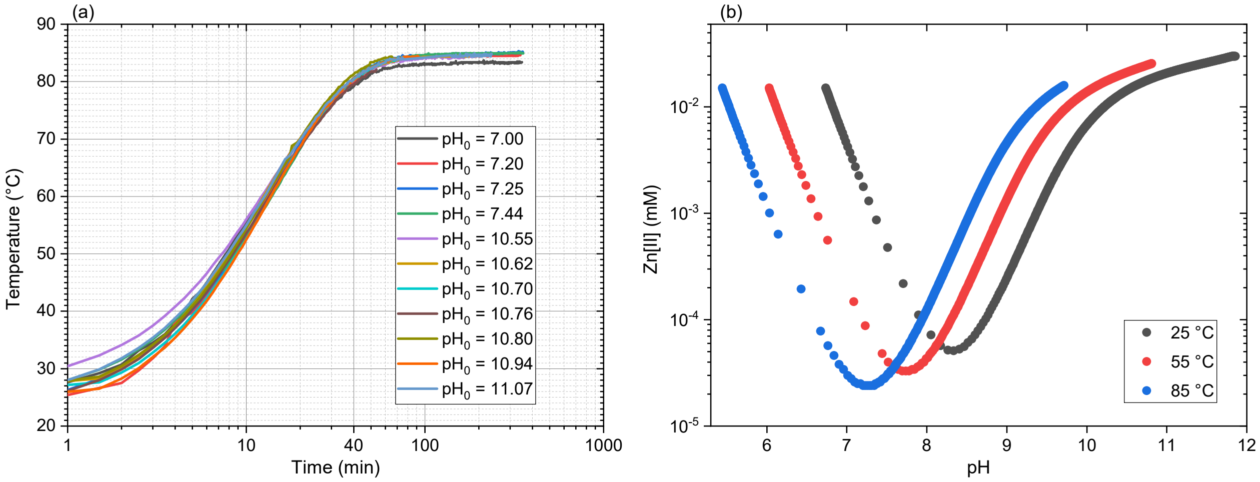

Fig. S3 (a) In situ measurement of temperature during the chemical bath deposition (CBD) of ZnO NWs grown with pH_0 values in the range of 7.00 – 11.07. (b) Theoretical solubility plot of Zn(II) species at different temperatures as a function of the calculated pH, obtained from thermodynamical calculations by Visual MINTEQ.

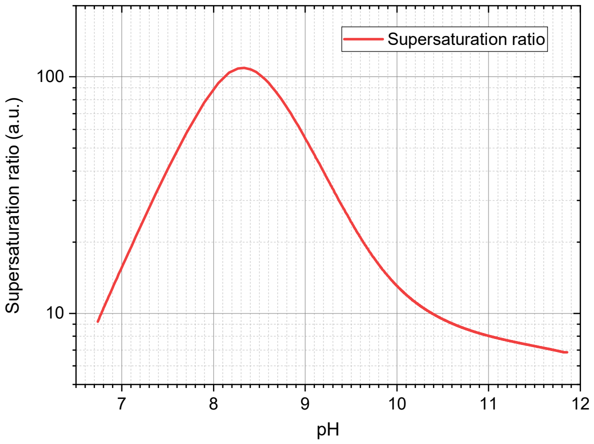

Fig. S4 Theoretical supersaturation ratio of Zn(II) species as a function of the calculated pH, obtained from thermodynamical calculations by Visual MINTEQ.

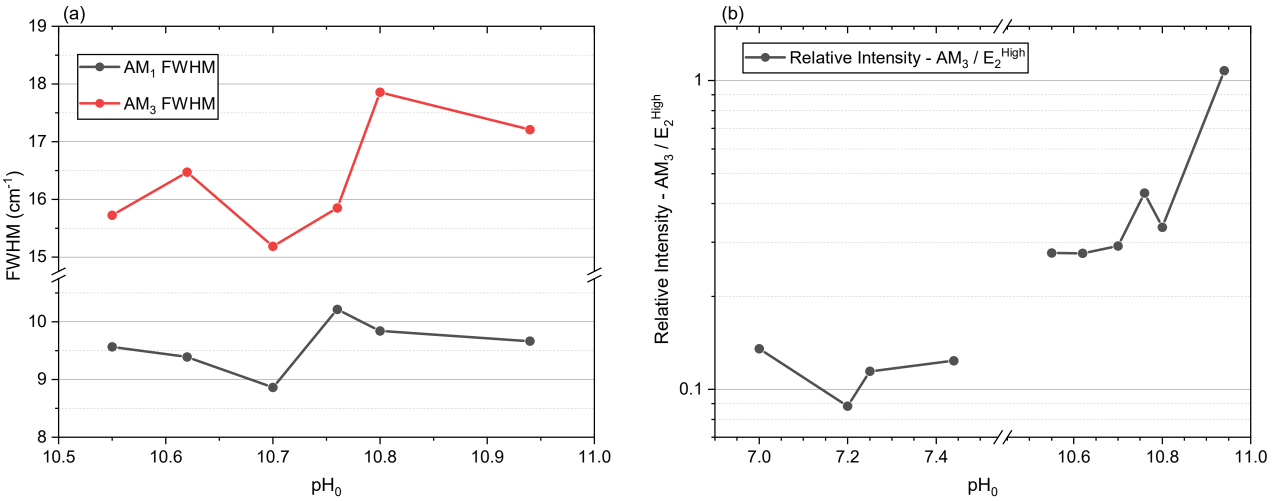

Fig. S5 Evolution of the (a) FWHM of the AM_1 and AM_3 modes, and (b) relative intensity $\left(\frac{AM_3}{E_2^{high}}\right)$ for the annealed ZnO NWs series grown by chemical bath deposition (CBD) with pH_0 values in the range of 7.00 – 10.94.

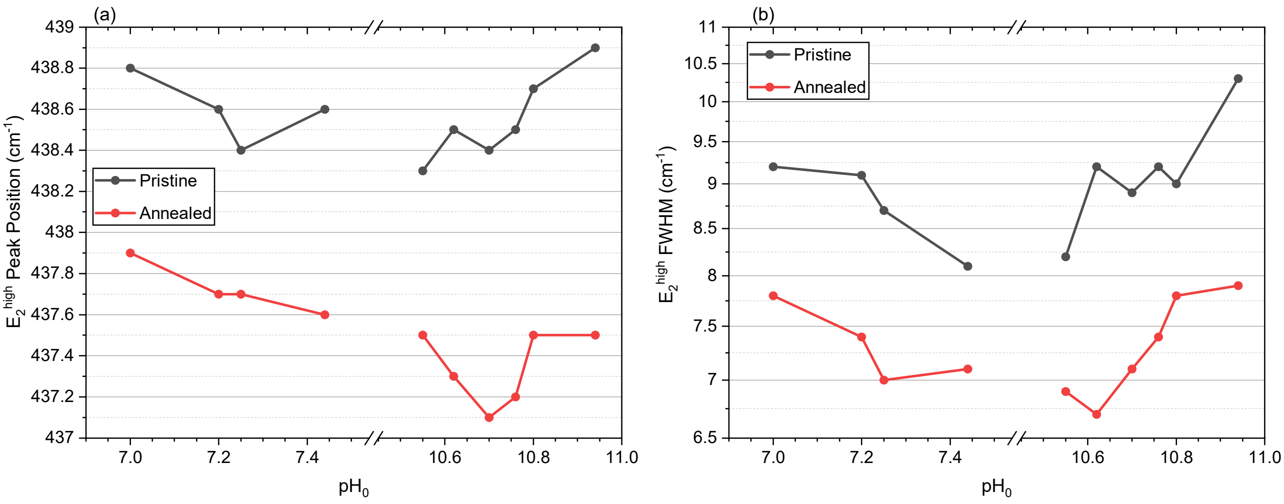

Fig. S6 Evolution of the (a) peak position, and (b) FWHM of the E_2^{high} Raman mode of ZnO NWs grown by chemical bath deposition (CBD) with pH_0 values in the range of 7.00 – 10.94.

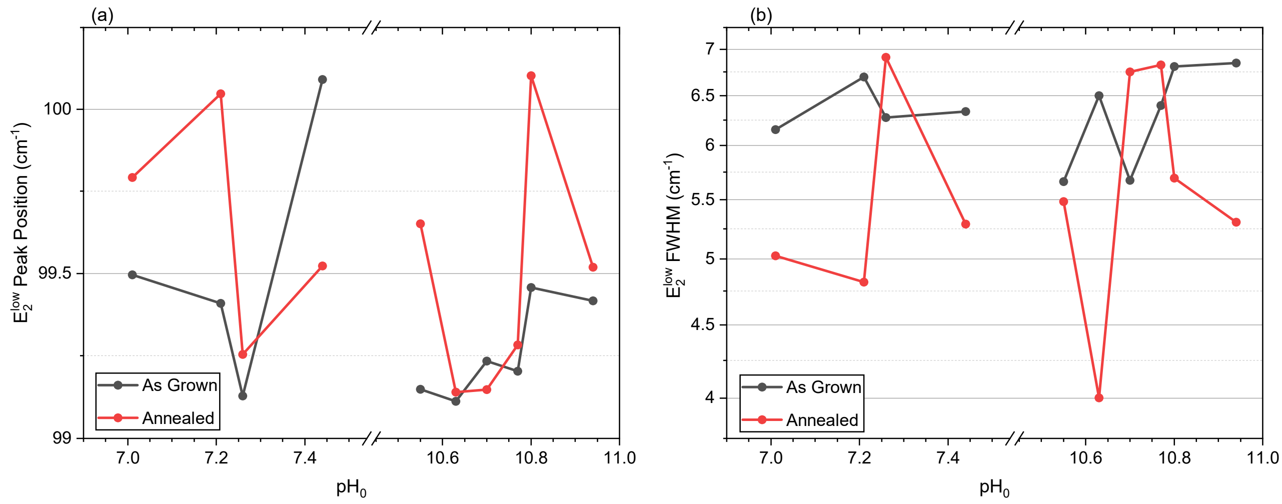

Fig. S7 Evolution of the (a) peak position, and (b) FWHM of the E_2^{low} Raman mode of ZnO NWs grown by chemical bath deposition (CBD) with pH_0 values in the range of 7.00 – 10.94.

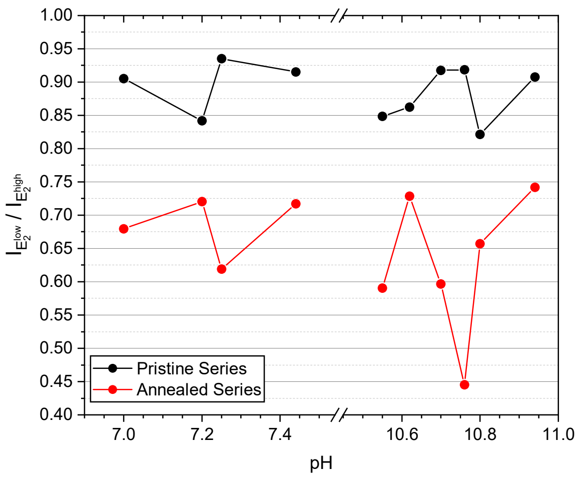

Fig. S8 Intensity ratio of E_2^{low} and E_2^{high} Raman modes of ZnO NWs grown by chemical bath deposition (CBD) with pH_0 values in the range of 7.00 – 10.94.

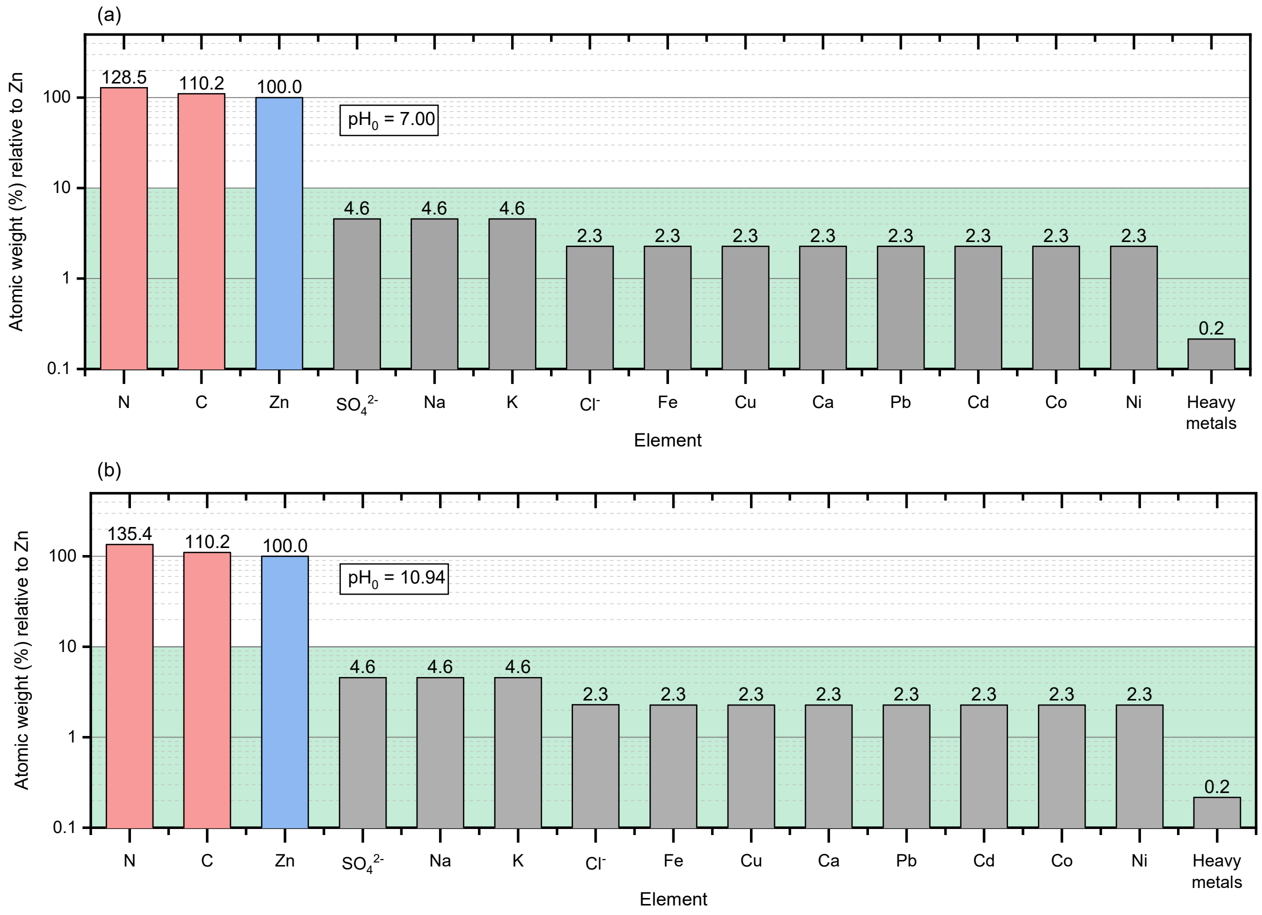

Fig. S9 Atomic weight (%) relative to Zn atoms of the foreign elements present in the chemical bath deposition (CBD) reaction for the growth condition of ZnO NWs at: (a) pH_0 of 7.00 and (b) pH_0 of 10.94. They come from the residual impurities presented in the Certificate of Analysis of the precursors:

$Zn(NO_3)_2 \cdot 6H_2O$ (Sigma-Aldrich), HMTA – $C_6H_{12}N_4$ (Sigma-Aldrich) and Ammonium hydroxide solution – NH_4OH (Sigma-Aldrich).

References

1. Limpijumnong, S. & Van de Walle, C. G. Diffusivity of native defects in GaN. *Phys. Rev. B - Condens. Matter Mater. Phys.* **69**, 035207 (2004).
2. Qian, G. X., Martin, R. M. & Chadi, D. J. First-principles study of the atomic reconstructions and energies of Ga- and As-stabilized GaAs(100) surfaces. *Phys. Rev. B* **38**, 7649–7663 (1988).
